# Regulation of acid-sensing ion channels by protein binding partners

**DOI:** 10.1080/19336950.2021.1976946

**Published:** 2021-10-27

**Authors:** Megan M. Cullinan, Robert C. Klipp, John R. Bankston

**Affiliations:** Department of Physiology and Biophysics, University of Colorado Anschutz Medical Campus, Aurora, CO, USA

**Keywords:** Acid-sensing ion channels, stomatin, PSD-95, RIPK1, binding partners, accessory proteins

## Abstract

Acid-sensing ion channels (ASICs) are a family of proton-gated cation channels that contribute to a diverse array of functions including pain sensation, cell death during ischemia, and more broadly to neurotransmission in the central nervous system. There is an increasing interest in understanding the physiological regulatory mechanisms of this family of channels. ASICs have relatively short N- and C-termini, yet a number of proteins have been shown to interact with these domains both *in vitro* and *in vivo*. These proteins can impact ASIC gating, localization, cell-surface expression, and regulation. Like all ion channels, it is important to understand the cellular context under which ASICs function in neurons and other cells. Here we will review what is known about a number of these potentially important regulatory molecules.

## Introduction

Ion channels rarely operate in isolation. They are often part of large macromolecular complexes which can contain signaling proteins, regulatory molecules, scaffolding proteins, and even other ion channels. These protein interacting partners can impact the gating, localization, or expression of ion channels and understanding them is critical for understanding how ion channels function in real cells. There are countless examples of interacting partners for ion channels that are necessary parts of their physiological function. KCNE1 significantly slows the activation rate of KCNQ1 and shifts the voltage dependence of activation tuning the channel to act as a delayed rectifier required for repolarization in the heart [1]. In skeletal muscle cells, interaction between L-type calcium channels and the ryanodine receptor is required for excitation-contraction coupling [2]. Years of work on this complex has shown that these channels require a number of other proteins, including junctophilin and Stac3, to form the proper signaling complex to elicit muscle contraction [3]. Ion channel interacting partners can also change how these proteins are organized in excitable cells. In neurons, for instance, PSD-95 is critical for recruiting AMPA receptors into clustered active zones in the postsynaptic membrane [4]. Beyond localizing the receptor to the proper position in the synapse, PSD-95 may also be responsible for recruiting a number of other proteins to the channel complex including Shank3, GKAP2, and Homer 1a [4] .

For the family of pH-gated ASICs, we know comparatively little about the sort of macromolecular complexes that these channels form in mammals. The ASIC homolog MEC-4 from *Caenorhabditis elegans* has been clearly demonstrated to be essential for some types of touch sensation [5]. While the channel itself does not appear to be mechanosensitive, it becomes a touch sensor by co-assembling with a large cohort of other proteins which tethers it to regions on both the inside and outside of the cell [6]. These tethers can then push and pull on the channel to open it upon mechanical deformation [7].

ASICs have short N- and C-termini but a number of proteins are already known to interact at these sites. These interacting partners have a variety of effects on the channel and can impact gating, localization, and expression (Table 1). In addition to their interaction with ASICs, many of these proteins have been shown to interact with other critical membrane proteins, suggesting potential mechanisms for the incorporation of ASICs into larger signaling complexes. The overall deficiency in our understanding of the role ASICs play *in vivo* is in part due to a lack of understanding of the cellular context under which these proteins function. Here we will review what is known about a number of proteins thought to interact with ASICs.

**Table 1.** ASIC modulation by protein binding partners. Summary of the effects on the current, biophysical properties, and surface expression of ASICs

Binding Partner	ASIC Isoform	Interaction Site(s)	Current Response	Proton Sensitivity	Cell Surface Expression	τ_des_	notes	ref
**Stomatin**	1a	n.m.	↔ I_MAX_	↔	↔	↔	May not bind	^[16,17]^
	2a	n.m.	↔ I_MAX_	↔	↔	?	May not bind	^[16,17]^
	3	CT, TM1	↓↓ I_MAX_	↔	↔	↔		^[16–18]^
**STOML1**	1a	n.m.	↓↓ I_MAX_	n.m.	n.m.	↔		^[20]^
	1b, 2a	n.m.	↔ I_MAX_	n.m.	n.m.	↔		^[20]^
	3	n.m.	↔ I_MAX_	n.m.	n.m.	↓		^[20]^
**STOML3**	1a, 1b,2b,4	n.m.	n.m.	n.m.	n.m.	n.m.	IP and FRET with STOML3	^[25]^
	2a	n.m.	?	?	↔	↔		^[25]^
	3	n.m.	?	n.m.	n.m.	n.m.		^[107]^
**α-actinin 1**	1a	CT	↔ I_MAX_	↔	↔	?		^[35]^
**α-actinin 4**	1a	CT	↓ I_MAX_	↑	↔	?	↑↑ τ_rec_	^[35]^
**CIPP**	3	CT PDZ Motif	↑ I_MAX_	↑	↔	n.m.		^[36]^
**PSD-95**	1	No binding	↔	n.m.	n.m.	n.m.		^[59]^
	2a, 3	CT PDZ Motif	↓ I_MAX_	↔	↓	↔		^[59]^
**NHERF1**	3	CT PDZ Motif	↑ I_MAX,_ ↑↑ I_SUS_	↔	↑	↑		^[63]^
**NHERF2**	3	CT PDZ Motif	↑ I_MAX,_ ↑↑ I_SUS_	↔	n.m.	n.m.		^[63]^
**Lin-7B**	1a	n.m.	↑ I_MAX_	n.m.	n.m.	n.m.		^[59]^
	3	CT PDZ Motif	↑ I_MAX_	↔	↑	↔		^[59]^
**AP2**	1a	Proximal CT	↓ I_MAX_	n.m.	↓	↔		^[72]^
**PICK1**	1a	CT PDZ Motif	↑ I_MAX_	n.m.	?	n.m.	Facilitates PKC regulation	^[38–40,76]^
	2a	CT PDZ Motif	?	↔	n.m.	↔		
**AKAP150**	1a	CT	?	n.m.	n.m.	n.m.		^[78,81]^
	2a	NT, CT	n.m.	n.m.	n.m.	n.m.		^[81]^
**Annexin II light chain p11**	1a	NT	↑ I_MAX_	↔	↑	↔		^[84]^
**RIPK1**	1a	CT	n.m.	n.m.	n.m.	n.m.	Triggers ASIC-dependent cell death	^[49,52]^

**Symbols and Abbreviations**: Modest Increase (↑), Large Increase (more than 30-fold) (↑↑), Modest Decrease (↓), Large Decrease (↓↓), No Change (↔), Conflicting or unclear results (?), Not Measured (n.m.), Desensitization Rate (τ_des_), Desensitization Recovery Rate (τ_rec_), C-Terminus (CT), N-Terminus (NT), Transmembrane 1 (TM1), Peak Current (I_MAX_), Sustained Current (I_SUS_).

## Proteins that modify ASIC gating

### Stomatin

**Figure 1.**
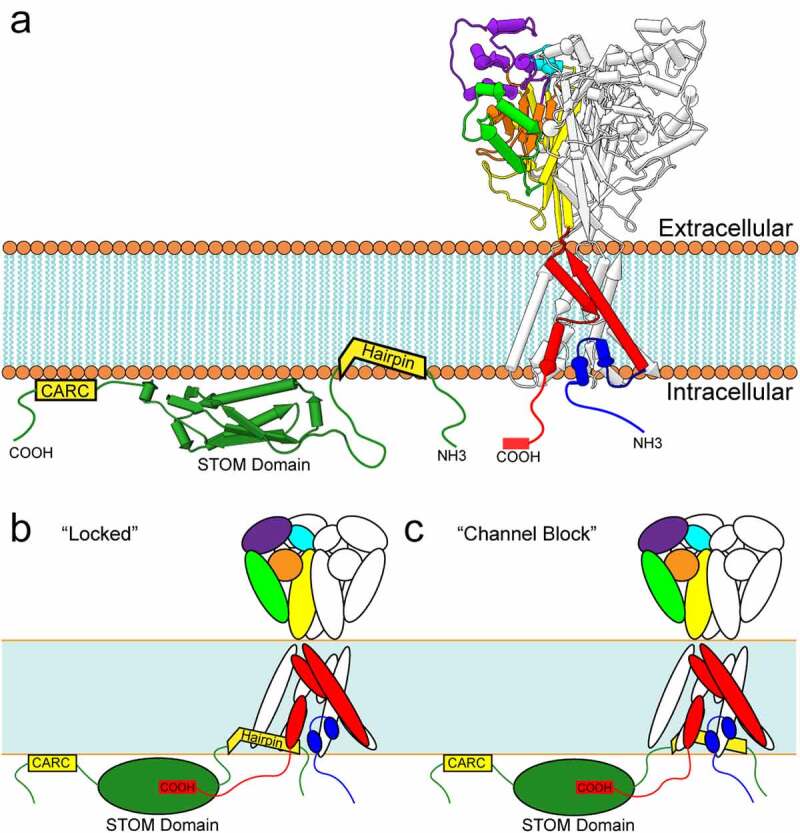
**Stomatin binding and inhibition of ASIC3**. A) Schematic representations of STOM and ASIC. (LEFT) Stomatin features a central STOM domain (green) (PDB Code: 4FVF). Depicted flanking the STOM domain are a CARC cholesterol-binding motif on the C-terminal end and an α-helical hairpin inserted into the membrane on the N-terminal end. (RIGHT) Structure of chicken ASIC1a in the desensitized state (PDB Code: 6VTK). Two ASIC monomers are shown in white and one monomer is colored to represent the major ASIC structural domains. N- and C-termini are depicted for one monomer as unstructured strands. B) Hypothesized interaction between STOM and ASIC3 shows a critical binding interaction between ASIC3’s C-terminus and the STOM domain, while a second interaction between the STOM hairpin and TM1 inhibits ASIC3 function by locking the channel in the closed or desensitized confirmation, or C) by blocking current flow. All structures shown in pipes and planks format using Chimera1.12

MEC-2, an integral membrane protein found in *C. elegans*, potentiates ASIC homolog MEC-4 mechanosensitive currents by ~40-fold and knockouts (KO) fail to respond to gentle touch [8,9]. There are a number of MEC-2 related homologues in mammals including stomatin (STOM), the stomatin-like proteins (STOMLs) 1–3, and podocin. Each of these proteins contains a STOM domain and are part of the larger SPFH (stomatin, prohibitin, flotillin, HflK/C) domain superfamily of proteins. STOM is a monotopic integral membrane protein consisting of a short intracellular N-terminus connected to a hydrophobic hairpin, followed by an intracellular C-terminus where the STOM domain is located (Figure 1a). In addition to the hairpin loop critical for membrane localization, STOM also has several palmitoylation and cholesterol-binding motifs important for membrane localization [10,11]. STOM domain proteins can be found throughout neurons in both the central and peripheral nervous system and are known regulate many membrane proteins including the glucose transporter GLUT-1, the anion exchanger AE-1, mechanosensitive PIEZO2 channels and many others [12–14].

While MEC-2 potentiates MEC-4/MEC-10 currents [15], STOM dramatically inhibits ASIC3 currents by nearly 200-fold [16,17]. This effect is isoform dependent as STOM has no impact on ASIC1a or ASIC2a current density. Recent work from our lab discovered two interaction sites for STOM on ASIC3 [17] . We found that the distal C-terminus (TCYLVTQL) was necessary for formation of the complex and subsequent regulation, while a second site on the first transmembrane domain (TM1) was required to confer functional regulation but not binding. Interestingly, the site on the distal C-terminus contains a PDZ recognition motif, which binds to a number of proteins that will be discussed in this review. STOM does not contain a PDZ domain; however, a hydrophobic pocket within the STOM domain which was previously shown to be important for ASIC interaction may serve a similar role as PDZ domains in interaction with ASIC3’s PDZ recognition motif [18]. Another group has suggested that a di-leucine in the proximal C-terminus may be important for STOM regulation as well [18]. A structure of the complex will be needed to determine the network of interaction between ASIC3 and STOM.

How STOM inhibits ASIC3 function is still unknown. However, a number of studies have shown that STOM inhibition of ASIC3 does not result from altered trafficking of the channel [16–18]. In addition, increasing the proton concentration does not overcome STOM inhibition which suggests that STOM does not act by shifting the pH dependence of channel activation [16,17].

It is possible that STOM acts by trapping the channel in the desensitized state. A point mutation in the extracellular domain of the channel, previously thought to eliminate desensitization, has been recently shown to, in fact, accelerate it [19]. However, this mutation does introduce a fraction of non-desensitizing channels that manifest as a steady state current. Our group recently showed that STOM could interact with this mutant channel without inhibiting the sustained current [17]. STOM, thus, could be acting by locking the channel in the desensitized state rendering the channel essentially nonfunctional (Figure 1b). It is still possible that STOM locks the channel in the closed state and simply prevents motions associated with gating. Given that an interaction with TM1 is necessary for STOM to inhibit ASIC3, the hairpin could also be positioned to interact with the newly uncovered reentrant loop and act as a blocker or cause a collapse of the selectivity filter in this region (Figure 1c).

### Stomatin-like proteins

Among the other members of the STOM family, stomatin-like protein 1 (STOML1) has also been shown to regulate ASICs in an isoform dependent manner [20]. When recombinantly expressed, STOML1 does not regulate ASIC2a or ASIC1b, but slightly speeds the desensitization of ASIC3 and drastically reduces acid-evoked currents of ASIC1a. The lack of regulation of ASIC1b points toward the N-terminus/TM1 as a critical regulatory site for STOML1 interaction with ASIC1a much like STOM regulation of ASIC3. Truncation of STOML1’s C-terminus was shown to eliminate inhibition of ASIC1a suggesting that this region is important for regulation. Interestingly, this truncation eliminated the sterol carrier protein-2 (SCLP2) domain unique to STOML1. Unlike STOM, which is found throughout neuronal subtypes, STOML1 is primarily found in the brain where ASIC1a is abundant [21–23].

Stomatin-like protein 3 (STOML3) effect on ASICs is less well understood. Initial observations showed that STOML3 was co-immunoprecipitated by ASIC2a, ASIC2b, and ASIC3 and that endogenous pH-gated currents were smaller in DRG neurons when STOML3 was present [24]. In addition, STOML3 appears to co-localize in vesicles within both CHO-K1 cells and DRG neurons [25]. STOML3 co-expression in CHO cells led to a relatively small reduction in ASIC2a and ASIC3 currents. STOML3 and STOM are 69% identical in humans, suggesting the possibility that the mechanism of binding and regulation might be conserved between these two proteins.

The physiological role of STOM’s interaction with ASICs remains unclear. STOM, STOML1 and STOML3 are all found alongside ASICs in the membrane of DRG neurons as well as in intracellular vesicle pools [20,25]. STOM and STOML KO mice show changes in acid-evoked currents as well as impaired touch sensation [24,26]. However, changes in touch sensation may partially reflect STOM-dependent effects on other proteins. For instance, PIEZO2 plays a known role in mechanosensation in primary sensory neurons and STOML3 is critical in PIEZO2 mechanosensitive gating [13,27,28].

### α-actinin

α-actinins help cluster membrane proteins, including ion channels, and signaling molecules into large complexes and can also link these complexes to the cytoskeleton [29]. α-actinins bind F-actin via N-terminal head domains. They have a central rod segment that is thought to be essential for interaction with membrane proteins and, lastly, they have two C-terminal EF-hand motifs. There are 4 α-actinin isoforms and α-actinin-1, α-actinin-2, and α-actinin-4 are all enriched at the postsynaptic membranes of dendritic spines [30]. α-actinins are critical regulators of ion channel gating and localization for a number of classes of ion channels including NMDA receptors, L-type Ca^2+^ channels, K^+^ channels, and TRP channels [31–34]. In addition, α-actinins have been shown to interact with ASIC1a both in a heterologous cell system as well as hippocampal neurons [35].

**Figure 2.**
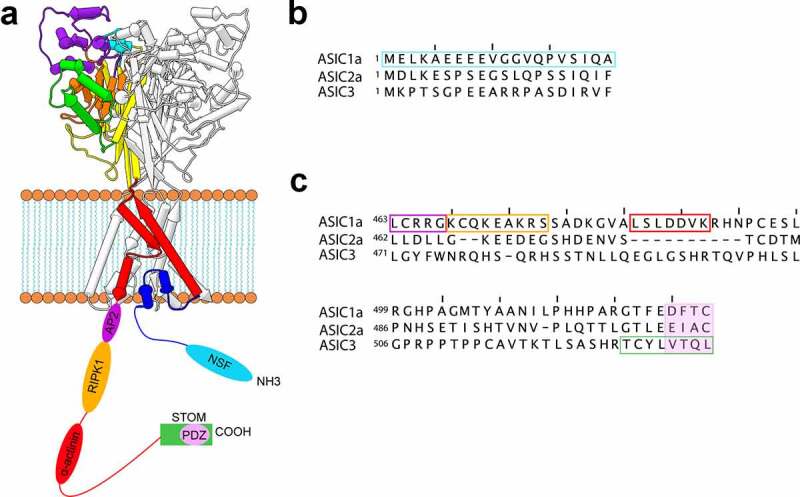
**Binding partner interaction sites on ASICs**. A) Schematic representations of ASIC with the N- and C-termini depicted as unstructured strands with the hypothesized binding sites for a number of binding partners shown. B) Aligned sequences of the N-termini of ASIC1a, ASIC2a, and ASIC3 from rat showing the putative NSF binding site in cyan. C) Aligned sequences of the C-termini of ASIC1a, ASIC2a, and ASIC3 showing putative binding sites for a number of proteins. The color of the boxes matches the color of the binding sites in panel A

The discovery of this complex was predicated on the observation that ASIC1a contained a putative α-actinin binding site (LSLDDVK) on its C-terminus (Figure 2). Both α-actinin-1 and α-actinin-4 can interact with ASIC1a while interaction between other ASIC isoforms and α-actinins does not occur [35]. Co-expression of α-actinin-4 decreased ASIC1a currents without an apparent change in surface expression. In addition, α-actinin-4 caused an alkaline shift in the pH dependence of channel activation and an acceleration in the recovery from desensitization [35]. This class of protein is a potentially interesting regulator of ASICs in that they may both tune ASIC gating as well as localize ASICs to key sites in neurons.

### CIPP

Channel-interacting PDZ domain protein (CIPP) is a four-PDZ domain containing protein known to interact with C-terminal PDZ recognition motifs of various channels [36,37]. CIPP is highly expressed in mouse kidney and brain tissue where it interacts with channels including KIR4.1 and NMDA receptors, in a PDZ domain-specific manner [37]. KIR4.1 surface expression is increased in the presence of CIPP which is mediated through an interaction with CIPP’s PDZ-2 domain while interactions with PDZ-3 domain were observed for the NMDA subunit receptor GluN2C [37].

CIPP has also been shown to have distinctive PDZ-domain interactions with ASICs [36]. PDZ recognition motifs are known to be present in ASIC1a, ASIC1b, ASIC2a, ASIC2b, and ASIC3 but the specific recognition sequences vary [36,38–41]. Deletion of either ASIC3’s PDZ recognition motif or CIPP’s PDZ-4 domain resulted in elimination of CIPP/ASIC3 interaction [36]. This interaction is specific to ASIC3 as neither ASIC1a, nor ASIC2a, were able to interact with CIPP. The functional consequences of this ASIC3/CIPP interaction is two-fold: Whole-cell current measurements of cells co-expressing ASIC3 and CIPP resulted in an alkaline shift in the pH dependence of activation and an additional ~5-fold increase in current magnitude at saturating pH [36]. Single-channel measurements indicate no changes in channel conductance suggesting that either a change in surface expression or open probability leads to the increase in current magnitude.

Physiologically, ASIC3 and CIPP have both been found together in DRG nociceptive neurons but consequences of CIPP/ASIC3 interactions *in vivo* remains to be elucidated [36]. The presence of four unique PDZ domains suggests that CIPP could help localize ASICs together with other channels or receptors in neurons. Additionally, the CIPP/ASIC3 interaction may be important in pathophysiological conditions, as increased expression of both proteins is observed in the presence of chronic hypoxia and elevated cytokines in rat chemosensory petrosal ganglion neurons [42].

## ASIC1a as a cell death receptor

### RIPK1

ASICs have long been thought to be vital in the process of cell death during stroke [43]. Tissue acidification occurs as a consequence of cerebral anoxia or ischemia. Acidification is thought to occur primarily due to buildup of lactic acid as a result of the switch to anaerobic glycolysis due to the lack of oxygen in the affected tissue [44]. During ischemic events pH can drop as low as 6 in the ischemic core and between 6.5 and 6.9 in the peri-infarct penumbra [45,46]. In several animal models of stroke, a decrease in tissue death has been observed in the presence of ASIC-blocking drugs/toxins, as well as in ASIC1a KO animals [43,47,48]. These observations led to the hypothesis that ASIC-mediated cell death during stroke arises from excitotoxicity caused by enhanced ASIC activity in the acidified tissue [43]. However, recent work has suggested that ASIC1a may act as a cell death receptor during stroke independent of its function as an ion channel [49].

It has been proposed that prolonged acidosis triggers a process called necroptosis during ischemia [49]. Necroptosis is a programmed version of necrosis, characterized by plasma membrane rupture and spillage of the cellular contents. While there seem to be several ways to trigger the necroptotic signaling cascade such as tumor necrosis factor α or interferon γ, in each case the highly regulated necrosome must form. The necrosome requires receptor-interacting protein kinases, RIPK1 and RIPK3, as well as mixed-lineage kinase domain-like pseudokinase (MLKL) [50]. Cell death eventually occurs because phosphorylated MLKL oligomerizes and translocates to and permeabilizes the plasma membrane and organelles [51].

RIPK1 is a multidomain cytosolic protein. These domains include an N-terminal kinase domain, an intermediate domain, and a C-terminal death domain. Wang et al. suggested that the acidification of the extracellular solution induced a physical association between ASIC1a and RIPK1 which led to the phosphorylation of RIPK1 and the triggering of necroptosis [49]. This necroptosis-dependent cell death was observed in cortical neurons and CHO cells expressing ASIC1a or ASIC1b, but not in CHO cells expressing ASIC2a or ASIC3. Cells did not undergo necroptotic cell death when RIPK1 inhibitor, Necrostatin-1 or ASIC inhibitors PcTx1 and amiloride were added to the cells.

Interestingly, acid-induced cell death was independent of ASIC1a’s ion conducting function but depended critically on the presence of the C-terminus [49]. While the precise-binding site is unknown, a peptide of the proximal C-terminus (residues 463–476 on ASIC1a from human) was sufficient to activate RIPK1 and trigger cell death when added to mouse cortical neurons [49,52]. The same group has suggested that the conformational change that induces RIPK1 binding is an unbinding of the ASIC1a N-terminus from this C-terminal RIPK1 binding motif [52]. *In vivo*, following middle cerebral artery occlusion, which is a mouse model of ischemic stroke, microinjection of a peptide of ASIC1a residues 1–20 significantly reduced the infarct volume. The authors interpret this as the N-terminal peptide outcompeting RIPK1 for binding to the C-terminus of the channel.

In addition to potentially binding the C-terminal death motif at rest, the N-terminus 1–20 bound to another newly identified accessory protein, N-ethylmaleimide-sensitive fusion ATPase (NSF) under acidic conditions [52]. ASIC1a pulled down significantly more NSF when exposed to pH 6.0 than when exposed to resting pH 7.4. This effect was eliminated when the first 20 residues of ASIC1a were truncated. The authors suggest that NSF binds to the N-terminus and prevents rebinding to the C-terminus which leads to increased exposure of the RIPK1 binding site. NSF is a member of the AAA^+^ superfamily that is primarily involved in vesicle-mediated transport and membrane fusion events [53,54]. NSF assembles into a homo-hexameric ring and forms the 20S supercomplex for membrane fusion with soluble NSF attachment proteins (SNAPs) and soluble NSF attachment protein receptors (SNAREs) [55]. NSF has been shown to impact the cell-surface expression of a number of ion channels and transporters but its effect on ASIC1a has not been examined [56].

Overall, these data suggest a hypothesis for the mechanism of ASIC1a-mediated acidosis-induced cell death during extracellular acidosis. Under normal resting conditions, the N-terminus of ASIC1a binds to the C-terminus of ASIC1a within residues 463–476. In the presence of prolonged acidosis, like during ischemic stroke, the N-terminus unbinds from the C-terminus and binds NSF, exposing the C-terminal RIPK1 binding motif. Exposure of this motif recruits RIPK1 which subsequently becomes phosphorylated and triggers the initiation of necroptosis. Further testing of this hypothesis is needed, but regardless, these data point to ASIC1a as an important target for developing drugs that treat cell death in stroke and suggest this C-terminal complex as a new druggable site on the channel.

## Proteins that primarily alter ASIC trafficking and localization

### PSD-95

Postsynaptic density protein 95 (PSD-95) is an abundant protein found in excitatory synapses where it forms large scaffolding complexes controlling excitatory homeostasis [57,58]. PSD-95 helps control trafficking and localization of receptors in the synapse through direct and indirect interactions with its three PDZ domains. PSD-95’s functional importance for ASICs was first identified for ASIC3 where the two proteins were shown to interact via the PDZ recognition motif on ASIC3 [59]. Binding of the two proteins did not alter ASIC gating, but led to a ~5-fold decrease in ASIC3 current due to a decrease in ASIC3 protein on the plasma membrane and also led to clustering of the channel [59].

Similar to its effects on ASIC3, PSD-95 has been shown to reduce ASIC2a acid-evoked currents by decreasing its trafficking to the membrane, an effect dependent on the PDZ recognition motif in ASIC2a [60]. In hippocampal neurons PSD-95 was vital for localizing ASIC2a to dendritic spines. Mutation of ASIC2a’s PDZ recognition motif reduced ASIC2a in spines and increased its presence in dendritic shafts [61]. Interestingly, although ASIC1a and PSD-95 do not directly interact, PSD-95 still impacted localization of ASIC1a/2a heteromers [59–61]. Overexpression of PSD-95 led to increased ASIC1a localization in dendritic spines in WT neurons, but ASIC1a localization was not altered in ASIC2a KOs [61].

### NHERF1 and NHERF2

Na^+^/H^+^ exchanger regulatory factor 1 (NHERF1) is a scaffolding protein made up of two PDZ domains and a C-terminal ezrin-radixin-myosin- binding domain (EB). The PDZ domains enable NHERF1 to homodimerize or heterodimerize with other NHERF isoforms, as well as interact with an assortment of PDZ binding proteins. The EB allows NHERF1 to interact with the actin cytoskeleton [62].

ASIC3 was found to bind both NHERF1 and NHERF2 *in vitro* via an interaction between the PDZ recognition motif on ASIC3 and the first PDZ domain on NHERF [63]. Complex formation between NHERF1 and ASIC3 resulted in a significant increase in plasma membrane localization of both proteins and a ~ 7-fold increase in ASIC3 peak current. However, binding of both NHERF1 and NHERF2 increased the non-desensitizing, or sustained, component of the ASIC3 current by between 31- and 42-fold suggesting that both NHERF1 and NHERF2 have an impact on channel gating as well as surface expression. Furthermore, the rate of desensitization was modestly slowed by NHERF co-expression while the pH dependence of activation was unaffected.

NHERF1 has been shown to have roles in a diverse array of systems including the colon, the immune system, and the kidney [64–66]. Little is known about the role it plays in neuronal function centrally or peripherally. However, both NHERF1 and ASIC3, but not NHERF2 are expressed in DRG where the two proteins co-localize and NHERF1 appears to increase ASIC3 surface expression [63]. While the significance that a NHERF1/ASIC complex may play in neurons is unknown, a potential hypothesis is that NHERF1 binding to ASIC favors forward trafficking of the channel to the plasma membrane while also directly modulating channel function. Like PSD-95, it may also serve to localize ASICs to distinct microdomains in neurons.

### LIN-7B

Lin-7 is a family of scaffolding proteins essential for synaptic development and function [67–69]. The family consists of Lin-7A, Lin-7B, and Lin-7C. The overall architecture of this family is highly conserved. They contain an N-terminally located PDZ domain and C-terminally located L27 domain. Lin-7B interacts with ASIC3 via the C-terminal PDZ recognition motif on the channel [59]. Lin-7B has little effect on channel gating but was shown to increase the magnitude of the current ~8-fold due to a significant increase in cell-surface expression. Lin-7B also modestly impacted ASIC1a currents increasing them by ~3-fold. The mechanism of this increase was not investigated but is presumed to also result from an increase in channel expression on the plasma membrane [59]. Physiologically, ASIC3 and Lin-7B mRNA have been found together in neurons from DRG, brain and spinal cord, but the importance of the Lin-7b/ASIC3 interaction *in vivo* remains poorly understood [59].

### AP2

Endocytosis is a key mechanism that controls cell surface expression for many ion channels. Clathrin-mediated endocytosis internalizes cargo through clathrin-coated vesicles [70]. The cargo for this form of endocytosis is identified via binding to clathrin adaptor proteins [71]. Adaptor protein 2 (AP2), one of the best studied clathrin adaptor proteins, binds to the proximal C-terminus of ASIC1a at residues 463–467 (LCRRG) [72]. This complex results in constitutive endocytosis that is both clathrin- and dynamin-dependent and leads to reduced surface expression of ASIC1a. Pharmacological blockade of this pathway or shRNA knockdown of AP2 both result in an increase of channels on the membrane and a modest increase in current. Interestingly, inhibition of this pathway also worsened acidosis-induced death of cortical neurons suggesting a role for channel internalization in cell death during ischemia [72].

## Proteins that primarily alter ASIC regulation by signaling molecules

### PICK1

Protein interacting with C-kinase 1 (PICK1) is a multidomain protein containing a PDZ domain, a Bin/amphiphysin/Rys (BAR) domain and a coiled coil region that allows PICK1 to homomultimerize. PICK1 is a peripheral membrane protein that binds to the plasma membrane through its BAR domain [73]. It is also known to bind to protein kinase C (PKC) through an interaction between the PDZ domain on PICK1 and the catalytic domain on PKC [74]. The PDZ domain within PICK1 enables it to interact with over 30 different proteins that are primarily receptors, transporters and kinases, including several ASIC isoforms [75]. The PDZ domain of PICK1 binds the distal C-terminus of ASIC1 and ASIC2 but not ASIC3 or related αENaC or βENaC [40].

The functional and physiological role of the PICK1/ASIC complex is intricate and controversial. When ASIC2a and PICK1 were co-expressed in COS cells, there was no change in current amplitude, pH dependence or kinetics [39]. Yet, a separate study showed a modest increase in cell-surface expression when ASIC1a and PICK1 were co-expressed in HEK cells [41]. However, cortical neurons from PICK1 KO mice showed larger ASIC currents likely due to an increase in both ASIC1a and ASIC2a surface expression [39,76]. In pulmonary arterial smooth muscle cells, there appeared to be no change in ASIC1a trafficking due to PICK1 [77]. PKA phosphorylation of ASIC1a in hippocampal neurons reduced both PICK1 association with ASIC1a and ASIC1a/PICK1 clustering in synapses [78]. These results suggest that any PICK1 mediated effect on ASIC surface expression or localization likely requires the correct cellular context and other, as yet unidentified, proteins.

As noted above, PICK1 does not appear to alter ASIC function directly via its binding. However, PICK1 is critical for PKC mediated regulation of the channel. PKC stimulation of cells expressing ASIC2a alone led to only a modest increase in ASIC2a current amplitude, while PKC stimulation with co-expression of PICK1 and ASIC2a led to a large, ~3-fold potentiation of ASIC2a currents [39]. This effect was not due to a change in single-channel conductance or due a shift in the pH dependence of channel activation leaving open the possibility that a change in open probability or a change in surface expression causes this potentiation of ASIC2a current. Furthermore, in neurons isolated from PICK1 KO mice, stimulation of PKC had no effect on ASIC function, suggesting that PICK1 is a necessary bridge between PKC and ASICs [76].

While PICK1 is unable to bind to ASIC3, it has been shown to affect ASIC3 when heteromerized with nonfunctional ASIC2b [79]. Like ASIC2a, PKC stimulation of ASIC2b containing channels increased current amplitudes. However, PKC stimulation also shifted the pH dependence of activation toward more basic pH values. The effect of PICK1 on other combinations of heteromeric channels has not been investigated.

Additionally, PICK1 has been identified as a parkin substrate [80]. Parkin is an E3 ubiquitin-ligase which when mutated often leads to ubiquitination defects in Parkin substrates and ultimately Parkinson’s disease. Parkin binds to the PDZ domain of PICK1 and monoubiquinates it. Co-expression of Parkin with PICK1 and ASIC2a prevented PKC-dependent potentiation of channel currents [80]. Moreover, hippocampal neurons from Parkin KO mice displayed a large PKC-dependent potentiation of ASIC currents not observed for WT suggesting that Parkin suppresses PICK1-mediated PKC regulation of ASICs.

### AKAP150

Like many ion channels, there is evidence that ASICs interact with A-kinase anchoring proteins (AKAPs) [81]. AKAPs organize cell signaling events by directing anchored enzymes to specific molecular targets and subdomains [82]. AKAPs are best known for binding PKA, bringing it to targets of PKA phosphorylation, but they are also now known to bind a whole host of signaling molecules. AKAP150 has been shown to interact with ASIC1a and ASIC2a via pulldown and co-immunoprecipitation experiments [81]. In addition, AKAP150 co-localizes with ASIC1a and ASIC2a in clusters in dendrites of cortical neurons [81]. Inhibition of PKA binding to AKAP150 using a peptide competitor reduced ASIC currents in neurons suggesting that AKAP150 may participate in PKA regulation of ASICs. However, Leonard et al. showed that neither activation nor inhibition of PKA altered ASIC currents in hippocampal neurons even though the drugs did alter the amount of channel phosphorylation measured [78].

## Concluding remarks

Despite the relatively small size of the ASIC intracellular domains, there have already been many potential interacting partners discovered with binding sites on both termini (Figure 2, Table 1). This review did not include a number of putative binding partners whose functional effect is either modest or not well understood. This list of proteins includes PIST, annexin 2 light chain p11, and MAGI-1b as well as chaperones like Hsp90, Hsc70, Grp78, Calnexin, and Grp94 [59,83,84].

Why do these binding partners matter? The role ASICs perform in normal physiology is not well understood. ASIC1a, for instance, have an ~half-activating pH of 6.6 and do not even begin to activate until ~pH 7.0 [85,86]. Does the synapse ever reach pH 6.6 or even 7.0 during normal function? Outside of pathophysiological conditions like stroke, there is still a great deal of debate about the pH of the synapse. Some specialized synapse like retinal bipolar cells have been estimated to acidify to pH as low as 6.9 [87]. However, a great deal of evidence has suggested that many synapses may actually alkalinize during activity [88–90]. Since synapses may only experience small acidification or even alkalinization, it is not clear how ASICs are activated.

Could some of these binding partners help explain how ASICs function in neurons? There are a number of potential ways in which this could happen. First, several of these proteins including CIPP and α-actinin-4 sensitize ASICs to protons allowing for activation under less acidic conditions.

Second, many proteins that interact with ASICs are known to organize larger signaling complexes. PSD-95 is known to be a major structural component of the synapse and critical for the proper organization of molecules in the synapse [91,92]. A typical post-synaptic density may contain 200–300 molecules of PSD-95 which help organize a huge variety of molecules in the synapse including NMDA receptors, AMPA receptors, Stargazin, and potentially ASICs [93,94]. Similarly, α-actinin can bind both NMDA receptors and ASICs which are both present in dendritic spines [60,61]. ASICs have been shown to be functionally coupled to GABA receptors, NMDA receptors, Slo1 Bk channel, AMPA receptors, and P2X channels [95–100]. ASICs have also been shown to be functionally coupled to GPCRs like P2Y_2_, μ-opioid receptors, 5-HT_2_ receptors and the CB-1 cannabinoid receptor [101–104]. Regulation by P2Y_2_ and 5-HT_2_ involve PKC phosphorylation and require PICK1 binding to ASICs [101,102]. It will be important to understand what sort of localized signaling domains are formed in different cell types and how these domains impact ASIC activity.

Finally, it is possible that, while the synapse does not significantly acidify, near the channel the local concentration of protons is higher than can be seen using the optical techniques that have made the bulk of the synaptic pH measurements. One way this could be achieved is through ASIC coupling to a proton source that could be an ion channel, transporter, or pump. The Na+/H+ exchanger has been implicated in synapse acidification in primary cortical neurons and is known to interact with NHERF and α-actinin [66,105,106].

This review highlights many protein partners of ASICs, but despite a growing body of knowledge of what proteins can bind to ASICs and what functional changes they elicit, a great deal more work is needed to understand the role these complexes perform in native tissues. ASICs are understood to impact pain sensing peripherally as well as memory, fear conditioning, panic, and anxiety centrally. However, we do not yet know if, for instance, PSD-95 trafficking of ASICs to synapses impacts any of these processes or if STOM expression and binding helps tune the sensitivity of peripheral neurons to ASIC-dependent pain sensing. Uncovering new modulators of ASICs and better studying the role these complexes play in real cells will be important for understanding the cellular context under which these channels function and allow us to better understand the physiological roles of ASICs.
